# Supplementary material for: Impact of dual-layer solid-electrolyte interphase inhomogeneities on early-stage defect formation in Si electrodes
Source: Nat Commun. 2020 Jul 1;11:3283. doi: 10.1038/s41467-020-17104-9 (PMC7329811; doi:10.1038/s41467-020-17104-9)
Supplement: Supplementary file 1 — Supplementary Information [file 41467_2020_17104_MOESM1_ESM.pdf]

Supplementary Information

Impact of dual-layer solid-electrolyte interphase inhomogeneities on early-stage defect formation in Si electrodes

Chen et al.

Supplementary Figures

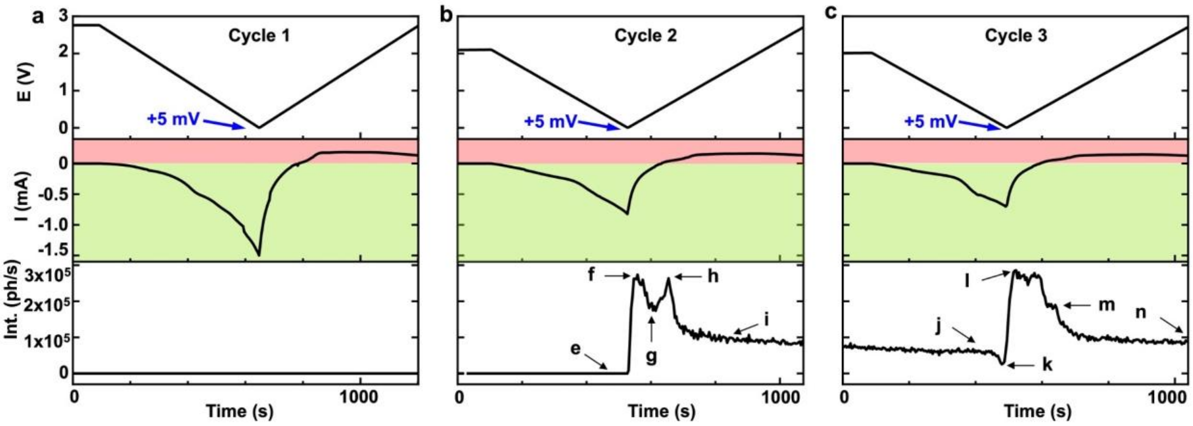

Supplementary Figure 1: Evolution of the total scattered intensity. *Potential (E), current (I) and total scattered intensity (Int) of the entire area (100×430 μm^2) respectively for the 1st (a), 2nd (b) and 3rd (c) cycle. The annotations (e-n) are the same as in Fig. 2. The light green and light red background in (a) to (c) illustrate the current for lithiation (negative) and delithiation (positive), respectively. No defect was observed and Int remained 0 during the entire first cycle.*

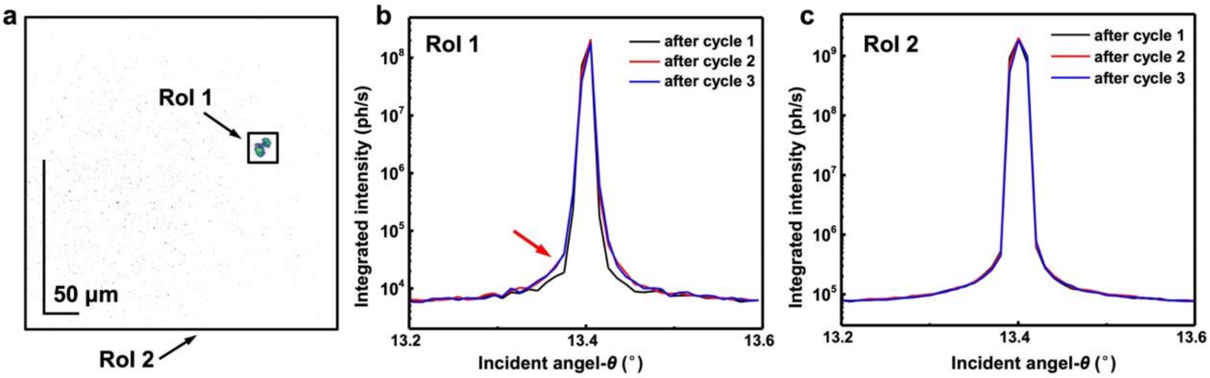

Supplementary Figure 2: Sensitivity of the FFDXM*. Definition of the RoIs (a). Rocking curve line profile generated by integrating the intensity in RoI1 (b) and RoI2 (c) after cycle 1, 2 and 3..*

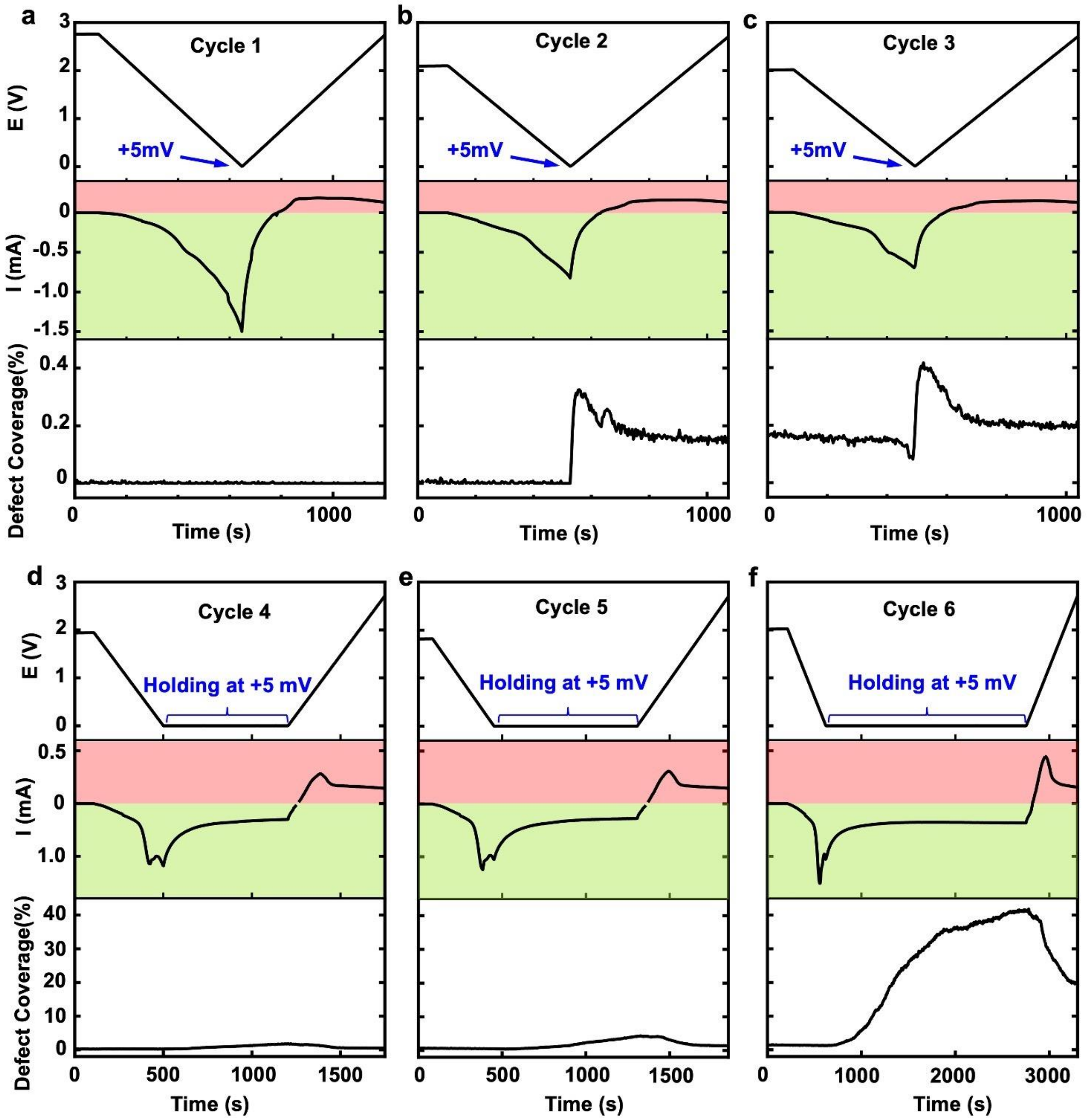

Supplementary Figure 3: Evolution of the surface area covered by the defects. *The potential, current and defect coverage are shown respectively for cycle 1 (a) to 6 (f). The result echoes what was shown in Supplementary Figure 2. The early-stage defects covered a mere 0.3% of the entire surface area (<100 ppm of the volume considering an X-ray penetration depth of 200 μm) and would hence be undetectable to non-local techniques such as conventional XRD. The defect coverage only became significant after 2000 s of accumulated holding at +5 mV. A surveyance scan covering 2×1 mm^2 of the Si electrode shows that at the end of the 6th cycle, about 20% of the surface was consistently covered with these defects.*

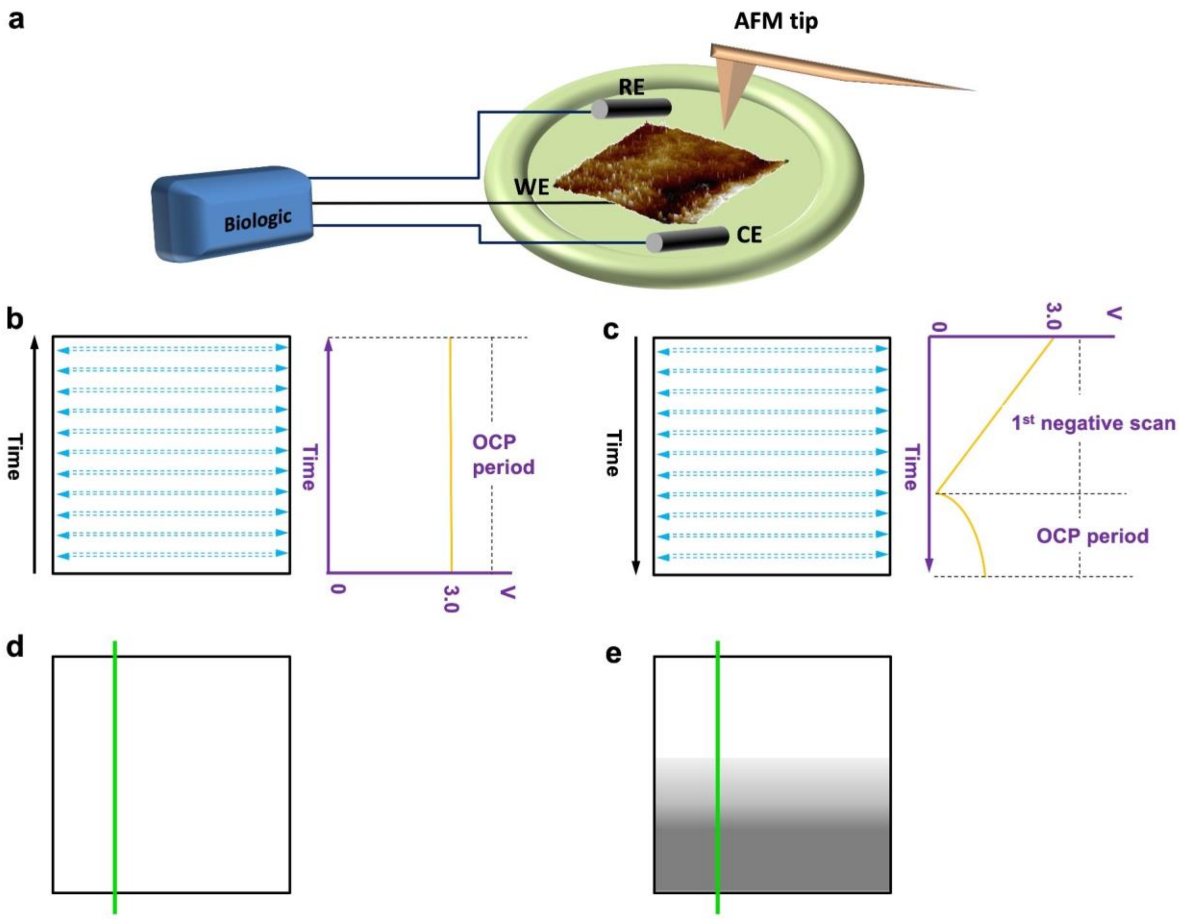

Supplementary Figure 4: Principle of operando AFM. *Experimental setup (a), showing a single-crystal Si sample, acting as the working electrode (WE), two lithium foils acting as the reference (RE) and the counter electrode (CE). The electrodes are assembled in a home-made AFM cell made of Teflon, which is inert with respect to the used electrolyte. Electronic feedthroughs facilitate the connections between the electrodes and the potentiostat. Operation principles of operando AFM during OCP (b) and potential scan (c) periods. During operando AFM measurements the whole surface is scanned in the trace-retrace manner, as indicated by the back-and-forth blue arrows. The general direction of the tip movement (black arrow) can either be from down to up (b) or from up to down (c). During scanning the electrochemistry information is simultaneously recorded, allowing plotting of for instance the voltage (orange line) of the sample during each line-scan. (d) where the height information of the surface was extracted for pristine Si (Supplementary Figure 5). (e) where the height information of the surface was extracted after scanning from* E_{ocp} *to +5 mV in the first CV cycle (Fig. 5a). The net height change (Fig. 5q) of the inner-SEI was obtained by subtracting the height of the surface of pristine Si (green line in Supplementary Figure 5) from the height of the surface covered with inner-SEI (green line in Fig. 5a).*

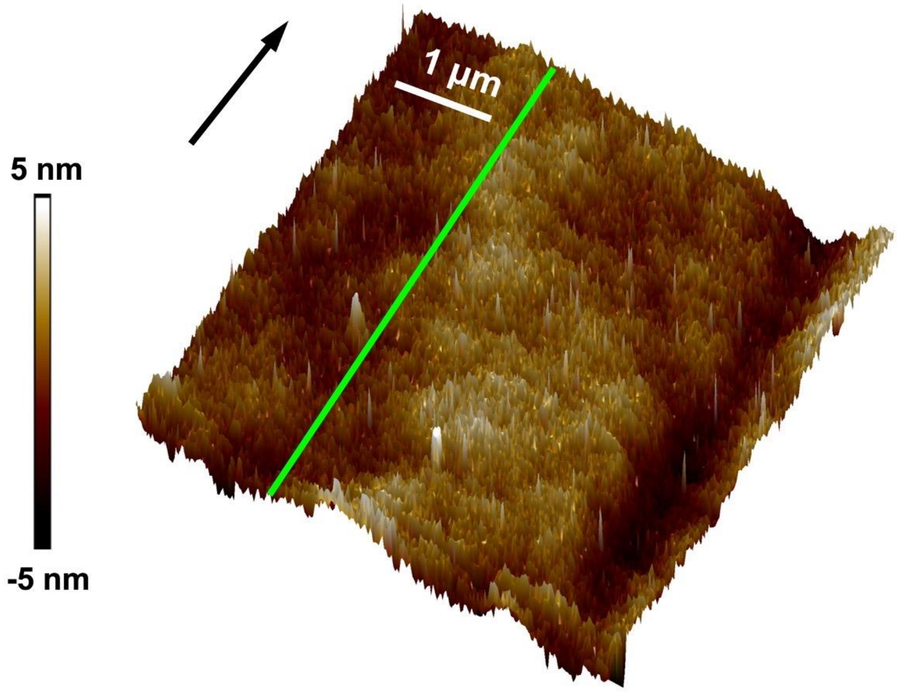

Supplementary Figure 5: Topography of the pristine single-crystal Si. *The topography image was measured during the initial open-circuit potential period.*

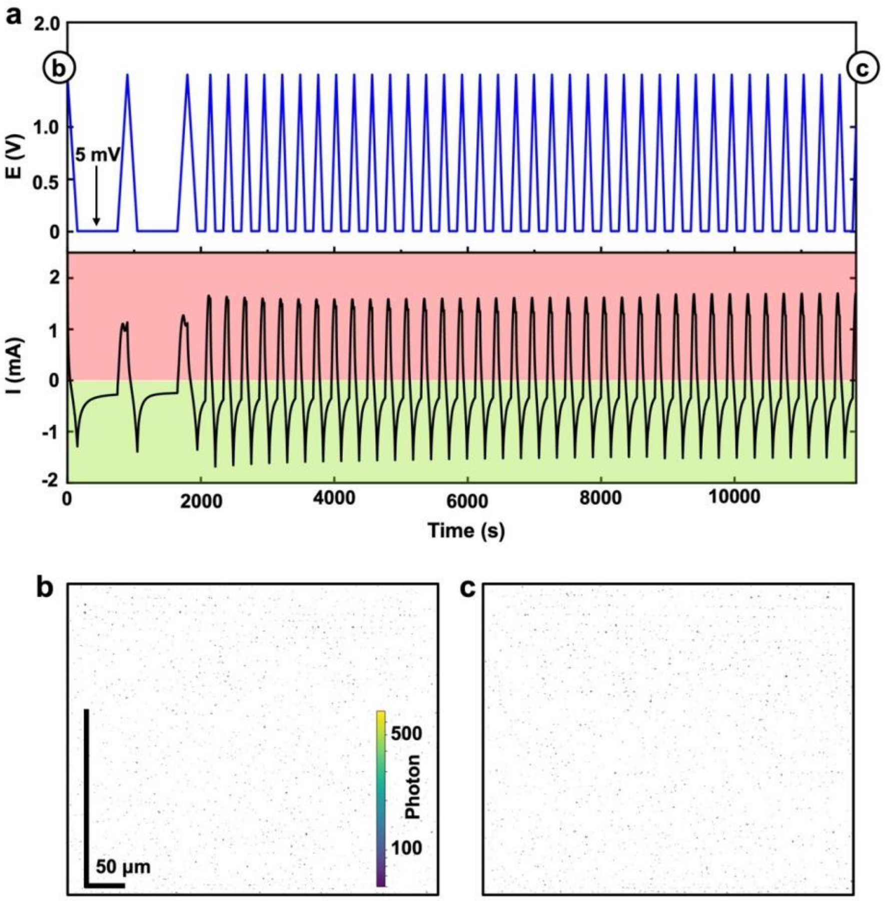

Supplementary Figure 6: Result on homogeneously deposited artificial-SEI. *Potential and current plot (a). FFDXM image before (b) and after (c) cycling showed no defect on the Si electrode. The artificial SEI of* $Li_4Ti_5O_{12}$ *(20 nm)-*Li_3PO_4 *(200 nm) was deposited by magnetron sputtering.*

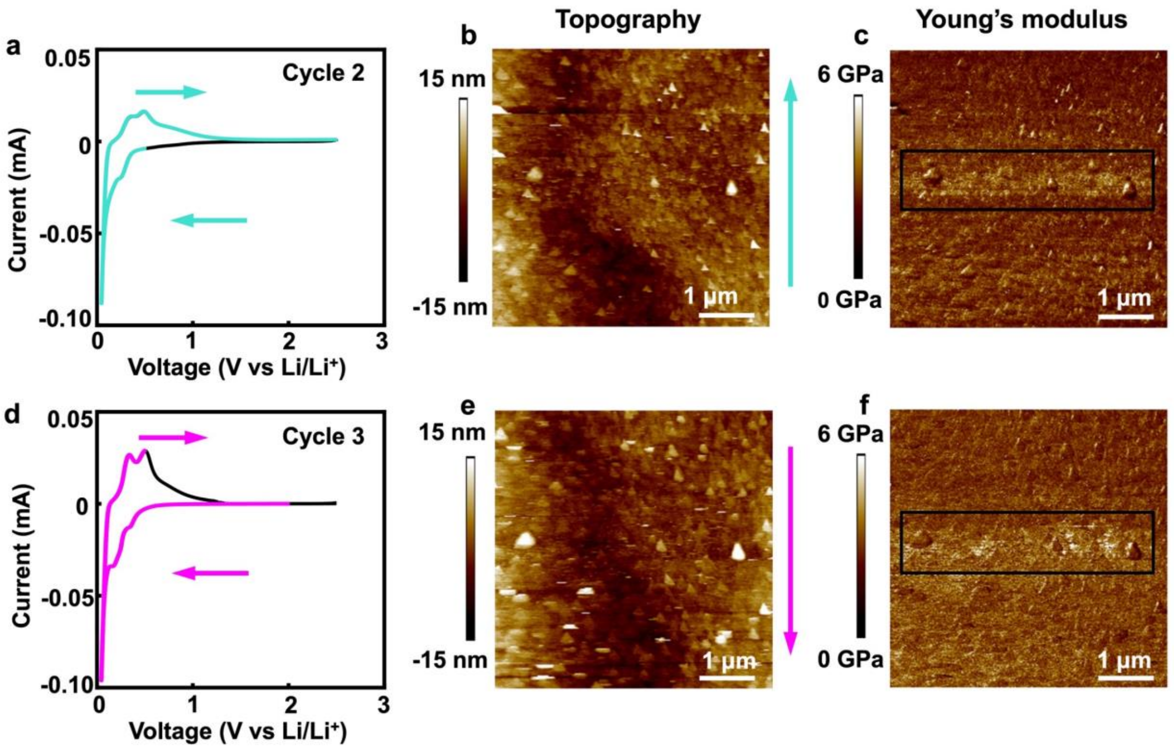

Supplementary Figure 7: Young's modulus mapping during cycle 2 and 3. *Topography and corresponding Young's modulus mapping of the single-crystal Si electrode during the 2nd (b and c) and 3rd CV-cycle (e and f). The corresponding CV potential range for each image is indicated by the colored curve for the 2nd (a) and 3rd (d) cycle, respectively.*

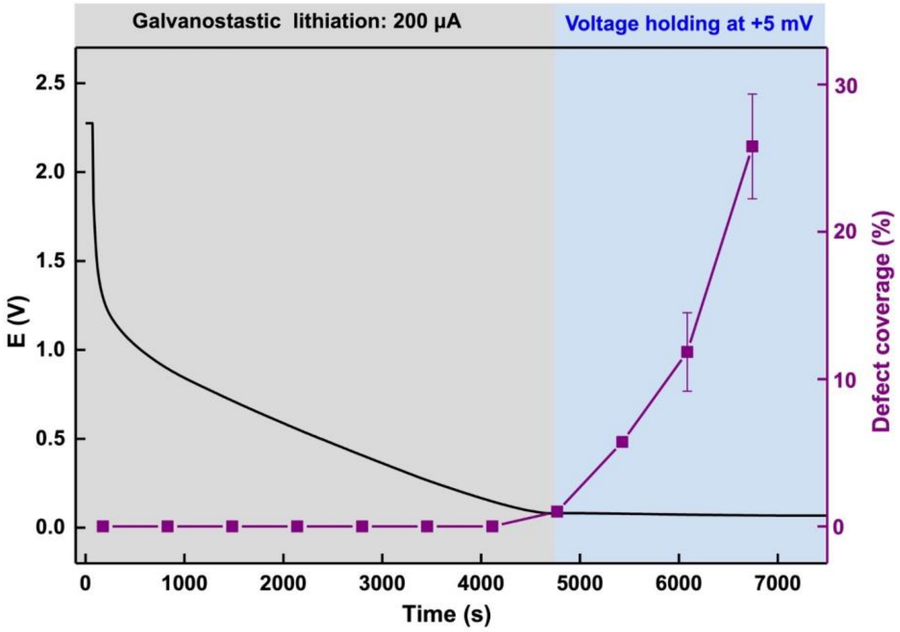

Supplementary Figure 8: Consistent behavior under a different cycling condition. *Si electrode potential (E) and percentage of the surface area covered by defects as a function of time during the 1*st *galvanostatic charging and constant voltage holding (+5 mV) of another Si electrode. The defect coverages were calculated from FFDXM measurements over an area of* 430×100 μm^2.

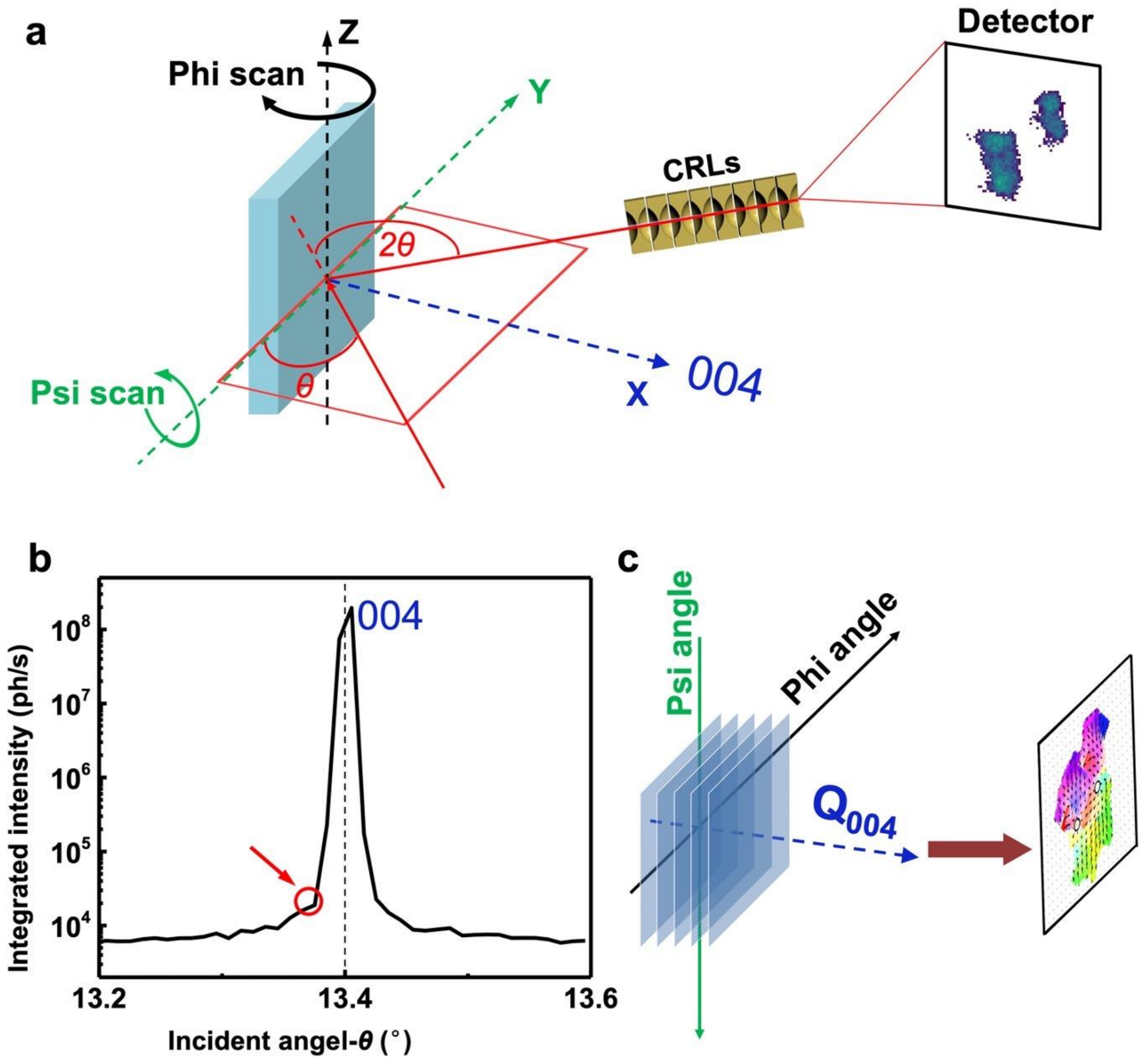

Supplementary Figure 9: Principle of FFDXM imaging. *Definition of the motors and the scattering angles (a). Integrated intensity of a rocking curve on a pristine Si electrode, the red circle marks the* θ *position for the time scan (b). Principle of 3D reciprocal space mapping (c).*

Supplementary Notes

Supplementary Note 1: To demonstrate that the early-stage defects would be essentially invisible for non-local techniques, we show results of rocking curves after the first 3 cycles (Supplementary Figure 2). The rocking curve line profile is generated by integrating the intensity over a selected region of interest (RoI) on the 2D detector. Two RoIs were chosen, RoI1 (8×25 μm^2) covers only the defective area while RoI2 (100×430 μm^2) covers the entire field of view (FoV), as shown in Supplementary Figure 2a.

The rocking curve line profile of RoI1 is different immediately after cycle 2. Higher intensity (marked by the red arrow in Supplementary Figure 2b) was observed at about -0.03° off the Si (004) Bragg θ angle. The higher intensity was due to the scattering by the defects, and it was at this angle where all the *operando* dark field imaging (time scan) were carried out. Meanwhile, no changes were observed on the rocking curve line profile of RoI2 for the first 3 cycles, see Supplementary Figure 2c. The integrated intensity over RoI2 represents what can be obtained with conventional XRD (one of the non-local techniques). While defects were already present inside RoI2 after the 2nd cycle, its integrated intensity showed little changes as it was dominated by scattering from the non-defective area. It is evident from this comparison that while conventional XRD is just as sensitive to lattice distortions as FFDXM, spatially resolved methods are required for detecting low density (early-stage) defects.
