# Supplementary material for: Impact of dual-layer solid-electrolyte interphase inhomogeneities on early-stage defect formation in Si electrodes
Source: Nat Commun. 2020 Jul 1;11:3283. doi: 10.1038/s41467-020-17104-9 (PMC7329811; doi:10.1038/s41467-020-17104-9)
Supplement: Supplementary file 3 — Description of Additional Supplementary Files [file 41467_2020_17104_MOESM3_ESM.pdf]

Description of Additional Supplementary Files

File Name: Supplementary Movie 1
Description: **Defect evolution during cycle 2.** Potential (E), current (I) and total scattered intensity (Int) curve during the time scan. The FFDXM images were taken at -0.03° off the Si (004) Bragg θ angle with a rate of 1 frame per second. Only area around the two early-stage defects is shown for simplicity.

File Name: Supplementary Movie 2
Description: **Defect evolution during cycle 3.** Potential (E), current (I) and total scattered intensity (Int) curve during the time scan. The FFDXM images were taken at -0.03° off the Si (004) Bragg θ angle with a rate of 1 frame per second. Only area around the two early-stage defects is shown for simplicity.

File Name: Supplementary Movie 3
Description: **Animation of SEI growth and early-stage defect formation.** Animated model which highlights the inhomogeneities of the dual-layer SEI and their impact on the formation of early-stage defects in the Si electrode.
